# Duplication of the *SLIT3* Locus on 5q35.1 Predisposes to Major Depressive Disorder

**DOI:** 10.1371/journal.pone.0015463

**Published:** 2010-12-01

**Authors:** Joseph T. Glessner, Kai Wang, Patrick M. A. Sleiman, Haitao Zhang, Cecilia E. Kim, James H. Flory, Jonathan P. Bradfield, Marcin Imielinski, Edward C. Frackelton, Haijun Qiu, Frank Mentch, Struan F. A. Grant, Hakon Hakonarson

**Affiliations:** 1 Center for Applied Genomics, The Children's Hospital of Philadelphia, Philadelphia, Pennsylvania, United States of America; 2 Division of Genetics and Department of Pediatrics, The Children's Hospital of Philadelphia, University of Pennsylvania School of Medicine, Philadelphia, Pennsylvania, United States of America; Ohio State University Medical Center, United States of America

## Abstract

Major depressive disorder (MDD) is a common psychiatric and behavioral disorder. To discover novel variants conferring risk to MDD, we conducted a whole-genome scan of copy number variation (CNV), including 1,693 MDD cases and 4,506 controls genotyped on the Perlegen 600K platform. The most significant locus was observed on 5q35.1, harboring the *SLIT3* gene (P = 2×10^−3^). Extending the controls with 30,000 subjects typed on the Illumina 550 k array, we found the CNV to remain exclusive to MDD cases (P = 3.2×10^−9^). Duplication was observed in 5 unrelated MDD cases encompassing 646 kb with highly similar breakpoints. *SLIT3* is integral to repulsive axon guidance based on binding to Roundabout receptors. Duplication of 5q35.1 is a highly penetrant variation accounting for 0.7% of the subset of 647 cases harboring large CNVs, using a threshold of a minimum of 10 SNPs and 100 kb. This study leverages a large dataset of MDD cases and controls for the analysis of CNVs with matched platform and ethnicity. *SLIT3* duplication is a novel association which explains a definitive proportion of the largely unknown etiology of MDD.

## Introduction

Major depressive disorder (MDD) is characterized by prolonged sadness and is often observed in conjunction with poor perception of self, frequent thoughts of suicide, lack of energy, and abnormal sleep [1]. MDD results in significant social, work/school, and overall health perturbation. The morbidity from depressive perceptions and mortality from suicide attempt are substantial. Genome-wide association studies (GWAS) have become a widely adopted methodology to scan across many observed variations known as single nucleotide polymorphisms (SNPs). Statistical association of SNP genotypes for differences in frequencies between population based case and control cohorts or family based linkage and transmission disequilibrium tests have been fruitful for a variety of disease phenotypes. However, psychiatric disease GWAS studies have revealed relatively few robustly associated and replicated loci [2], [3], perhaps attesting to the more complex heterogeneity underlying psychiatric disease phenotypes.

Genome-wide association studies of SNP genotype frequencies in large MDD populations have revealed significance in *PCLO* [4], *ATP6V1B2* [5], *SP4* [5], *GRM7* [5], 3p21.1 [6], *GLYATL1* [7], and *RYR3* [7]. While additional and independent replication studies are awaited for some of these loci, it is noteworthy that these genes support the disruption of neurotransmission networks of the brain as the underlying cause of MDD. Genome-wide arrays provide both SNP genotype and intensity data which we have evaluated in tandem to assess the pattern and frequency of CNVs in a MDD case control cohort. CNVs were called in each individual and subsequently associated with the MDD phenotype, using a case control study design, to discover the most common CNV in cases which was exclusive when compared to a large control group.

## Results

We processed genotype and intensity data for all 599,164 probes of the Perlegen 600 K dataset for 3,761 MDD cases and controls, 1,682 Psoriasis cases and controls, and 2,788 ADHD trio samples available through dbGaP. We first converted the Cartesian coordinate values X and Y into Polar coordinates Theta and R which correlate clearly to genotype and intensity values. The observed values were then clustered to establish null expected values for Theta and R for AA, AB, and BB states. Clustering was successful on 586,730 probes. Values for B allele frequency (BAF) and log R ratio (LRR) were then calculated for each SNP in each sample based on this cluster. A HMM model was trained specifically for the resulting data using PennCNV [8]. The population allele frequency of each SNP was calculated to weigh the probability of observing homozygote genotypes. The SNP physical positions were given to weigh probability of extending CNV calls vs. boundary truncation. Following QC for genotypes successfully called and exclusion of any poor quality DNA and ethnicity outliers as described in Methods, we defined a clean data set of 1,693 MDD cases and 1,697 controls at low probability for MDD, 1,600 controls from a publically available psoriasis case:control study in dbGaP, and 1,209 parents from a ADHD parent-offspring project also in dbGaP. This gave us a total size of 1,693 MDD cases compared to 4,506 controls all typed on the Perlegen 600 k array. The MDD case and controls that were at low probability for MDD were clustered together yielding 97.9% of SNPs with acceptable clustering. However, the samples were not required to have high SNP call rate since duplications and homozygous deletions can deviate from the three expected diploid genotype modes in an informative manner. The psoriasis similarly yielded 95.0% of SNPs usable while ADHD parents had 64.6%. Therefore, the rates of exclusions for poor sample quality and individual SNP quality were highly similar for cases and controls with exception of the ADHD parents. The higher drop-out rate for ADHD parents may represent a more unfiltered data release than the other projects, more variability in sample quantity, less ability to rerun failing samples, or other technical variability. Nonetheless, the MDD cases, MDD screened controls, and psoriasis controls showed similar exclusion rates. Similarly, the genomic inflation factor of the overall dataset was low showing that variability from ancestral differences and technical artifacts were minimal.

The Perlegen data has proven to have higher noise content than comparable genome-wide SNP arrays from Illumina and Affymetrix. The essential chemistry problem which dilutes CNV signal in Perlegen 600 k data is that PCR is run to the point of saturation. Long-range PCR products are pooled together to facilitate probe binding and boost signal for each SNP. However, as a result the relative differences in intensity for different regions are diminished. The problem is that many CNV calling algorithms use intensity alone, rather than intensity and genotype in tandem as PennCNV does. While there is a higher noise content to the data, we trained a HMM specifically for this data and we have visually validated all CNVs called by the Perlegen 600 K platform [9]. We also observed inheritance of CNVs at loci across the genome which boosted confidence in CNV calls.

We used PennCNV-Affy which evaluates intensity in terms of Log R Ratio rather than Log 2 Ratio. The Log 2 Ratio is based on quantile normalization, which derived from the sum of signal intensity for A allele and B allele for each sample, the median across all samples, and for a given sample, the A+B allele intensity is divided by the median value and logarithm base 2 applied. In contrast, the Log R Ratio is based on defined signal intensity clusters of AA, AB and BB genotypes across a large group of samples. Given this expected intensity value, the observed A+B signal intensity data is divided by this expected value, and the logarithm taken.

We evaluated the SNP-based frequency of deletions and duplications between cases and controls to identify CNV regions (CNVRs) common to cases and not observed in controls (Table 1). We evaluated all 43,309 case and 89,744 control CNV calls generated. We also evaluated only calls with at least 10 SNPs and 100 kb. The case and control cohorts lowered to 647 case and 1,590 control samples with 921 and 2,594 respective CNV calls meeting this size condition. Certainly evaluating all called CNVs regardless of size will introduce a substantial proportion of false positive calls. However, comparison of resulting loci based on the conservative filter and the all-inclusive filter is necessary to see if any controls impact the locus with calls below the threshold. In this way, the true exclusivity of case calls can be evaluated. We evaluated all associated CNV regions recurrent in MDD cases and exclusive with respect to controls (Table 1). Conversely, we did not observe any CNV regions recurrent in controls not observed in cases.

**Table 1:** All associated CNV regions recurrent in MDD cases.

CNV Region	Count SNPs	Count MDD Cases	MDD Case IDs	Gene(s)
chr5:168423758-169070607	198	5	05D01518 06D02197 06D06042 06D06073 06D11362	*SLIT3,CCDC99,DOCK2*
chr5:115261015-115369537	54	3	06D00054 07D00185 07D00852	*AP3S1,AX747550,FLJ90650*
chr5:25121416-25298271	18	3	06D03270 06D04240 06D05804	*AK309747/CDH10* *
chr4:65074807-68777792	273	2	05D00964 05D02984	*EPHA5,CENPC1,STAP1,UBA6,GNRHR,TMPRSS11*
chr15:97399748-97770838	95	2	06D01590 06D01080	*SYMN, LRRC28,TTC23*
chr10:81631178-81930681	68	2	05D02768 07D00173	*SFTPD, C10orf57, PLAC9, ANXA11*
chr8:21188347-21318056	64	2	05D02193 05D04794	*AK057515/GFRA2* **
chr7:17201925-17473214	62	2	06D02134 06D06089	*AHR*
chr7:8241584-8363438	42	2	05D03460 05D04353	*ICA1,AX746880,CR624517*
chr7:24922773-25040936	41	2	05D00474 06D00295	*OSBPL3*
chr1:239103312-239231293	35	2	05D01560 06D01602	*RGS7*
chr11:108105203-108301884	12	2	06D01991 07D00860	*DDX10*

Listed are all loci with CNVs of at least 10 SNPs and 100 kb that were observed to be recurrent among MDD patients and not found in any controls of substantial overlap. All CNVs happened to be duplications and directly impact the genes indicated except

*CDH10 which is proximal 440 kb and involved in axon outgrowth and guidance and

**GFRA2 which is proximal 275 kb and involved in neuronal survival and differentiation. Note also *ICA1* which is involved in neurotransmitter secretion. Given the PCR saturation of Perlegen 600 K processing, it is fitting that the intensity signal is diminished. However, the genotype signal is very clear and duplication calls leverage the AAB and ABB calls to deliver reliable calling. The observation of a run of homozygosity is quite common by chance so a lower intensity is important for deletion calls but diluted in this case.

**Figure 1:**
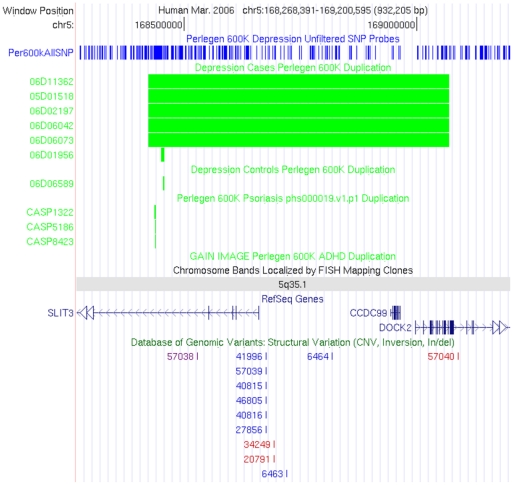
Duplication of 5q35.1 impacting *SLIT3* observed in 5 unrelated MDD cases. The coverage of SNPs on the Perlegen 600 K array is shown across the 5q35.1 locus with vertical blue lines. There are 198 SNPs within the duplication call boundaries shown as green rectangles for the 5 MDD cases with sample IDs listed to the left. Depression control, Psoriasis, and ADHD GAIN Perlegen 600 K sample sets are shown to not have CNV calls of the large size observed in MDD cases. All calls generated are shown for completeness of observation. Very small calls of 1.8 kb and less are observed in this region which is dissimilar from the case calls. The genes SLIT3, CCDC99, and DOCK2 are shown to have exons impacted by the duplication. Finally, the Database of Genomic Variants entries are shown which are very small and do not impact exons.

The CNV with the highest frequency in cases, exclusivity with respect to controls, and with clear duplication signals was a locus on 5q35.1 which impacts exons of *SLIT3*, *CCDC99*, and *DOCK2 (*P = 2.0×10^−3^). We ran permutation analysis through 10,000 simulations of case and controls to determine the probability of such an observation by chance (P = 2.7×10^−3^). Depression cases 05D01518, 06D02197, 06D06042, 06D06073, and 06D11362 were clearly scored with large duplication CNVs. The calls were near identical with coordinates on chr5 ranging from 168,423,758–169,070,607 shared by all 5 samples (Figure 1). There were 198 SNP probe signals in agreement of the duplication signal with SNP coverage nearby 5′ and 3′ breakpoints, providing clear support for the CNV call and accurately capturing the reproducible calling boundaries. The physical size is 646 kb which was well above our conservative threshold of 100 kb. The SNP based coordinates begin with rs4868223 and end with rs373808. The PennCNV confidence score was high at 276. The large region is very clear of any similar overlapping calls. The largest control CNV in this region encompassed only 1.8 kb overlapping the duplication observed in the depression cases. This low-confidence CNV call encompasses less than 0.3% of the associated region and does not impact genic exons and as such is an unimportant variation. There are similarly small and non-exonic entries in the Database of Genomic Variants (Figure 1). To evaluate the exclusivity of this duplication further, we evaluated 30,000 patients without neurological disorders typed on the Illumina 550 k platform and did not observe the duplication in these additional controls (P = 3.2×10^−9^).

**Figure 2:**
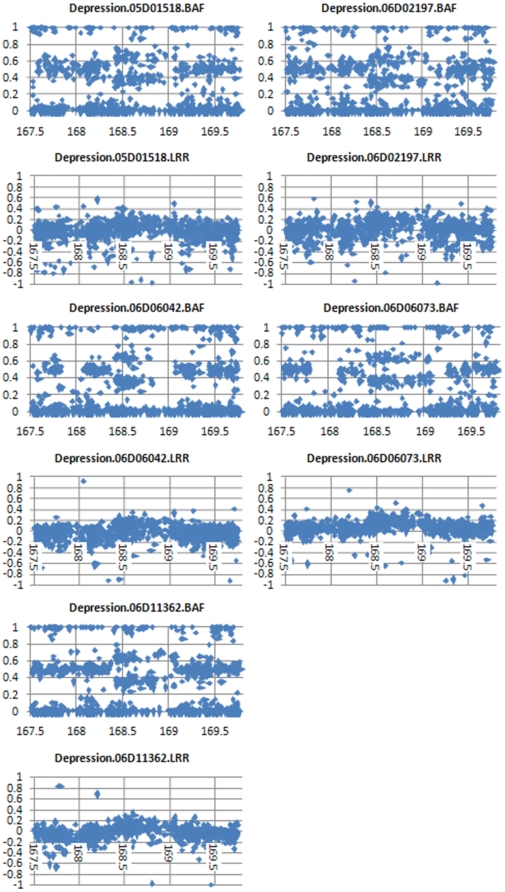
BAF and LRR SNP-based values for 5 MDD cases with Duplication of 5q35.1. The X axis represents the physical position on chromosome 5 in MB. BAF and LRR are derived values representing genotype and intensity content from the theta and R values of the raw Cartesian X and Y values provided directly by the Perlegen 600 K array. BAF and LRR values for 851 SNPs chromosome 5 ranging 167507516–169763949 are displayed to provide a 5′ and 3′ frame of normal diploid genome surrounding the duplication CNV for each sample.

**Figure 3:**
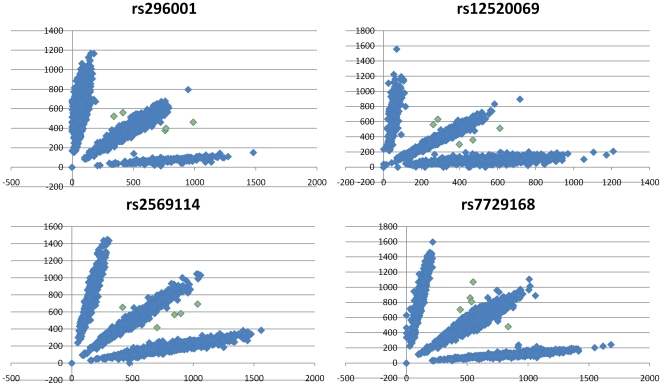
X and Y raw values showing common AA, AB, and BB states and rare AAB and ABB states. Raw Cartesian SNP Clusters with duplicated depression cases colored in green. Duplication AAB and ABB calls are found on multiple SNPs across the 5q35.1 region.

**Figure 4:**
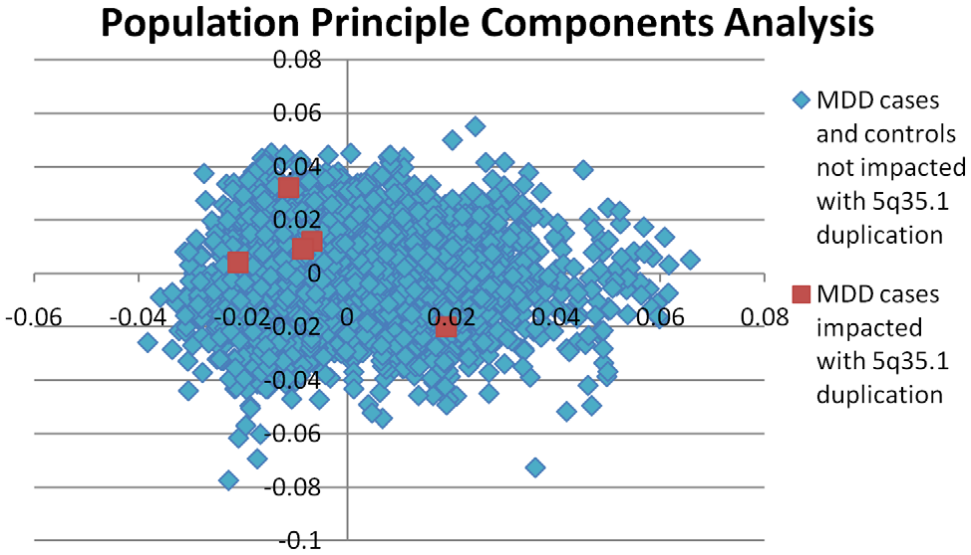
Eigenstrat Principle Components Analysis. The distribution of the second principle component is plotted versus the third principle component. The overall Caucasian population is shown to be homogenous to avoid spurious results arising from population stratification. The five cases with 5q35.1 are shown in red to show that they are representative of the overall population and not in any way outliers.

To gain further confidence in the duplication calls on 5q35.1, we extracted and viewed BAF and LRR values derived from the Perlegen 600 k data for the CNV region and flanking diploid regions (Figure 2). The BAF shows clear enrichment of values ranging 0.3–0.4 (AAB genotype) and 0.6–0.7 (ABB genotype) in the duplicated region. We observed an average of 83 SNPs throughout the duplicated region in these ranges which are all highly supportive of a duplication call. This clearly contrasts from the normal diploid signals shown 5′ and 3′ for each sample with many BAF signals ranging 0.4 to 0.6 (AB genotype). BAF values in this range make the duplication call very clear for both a HMM algorithm and a graphical review. The LRR data points are also elevated in an overlapping supportive manner. The majority of LRR signals are above the reference 0 expected diploid level and values above 0.2 are enriched. The LRR was especially important for sample 06D06042, which happened to have a large run of homozygosity which encompassed the 3′ end of the duplication and thus did not show AAB or ABB signals in this region. Furthermore, the raw Cartesian SNP clusters, which represent values completely unmodified by our methods, across the 5q35.1 region show clear AAB and ABB clusters (Figure 3). Although we have done pairwise genotype comparisons as an upfront filter to remove related individuals, we present the very low similarity values between these 5 cases to show these are indeed independent observations (Table 2). We also show these samples to be representative of the Caucasian cohort studied (Figure 4).

**Table 2:** SNP genotype similarity of 5 individuals with 5q35.1 duplication to prove unrelatedness.

ID1	ID2	PI HAT
05D01518	06D02197	0
05D01518	06D06042	0.01477
05D01518	06D06073	0.01027
05D01518	06D11362	0
06D02197	06D06042	0
06D02197	06D06073	0
06D02197	06D11362	0
06D06042	06D06073	0.0225
06D06042	06D11362	0.02282
06D06073	06D11362	0

Pairwise identity by descent (IBD) comparisons of 5 5q35.1 duplication MDD cases.

## Discussion

Our report represents the first genome wide association analysis of CNVs in MDD. The study cohort consisted of 1,693 MDD cases compared to 4,506 controls typed on the Perlegen 600 K platform. The most significant locus included *SLIT3*, *CCDC99*, and *DOCK2* on 5q35.1. Duplication was observed in 5 unrelated MDD cases encompassing 646 kb with highly similar breakpoints. While the Perlegen 600 k array has been deemed to be an array with data that is difficult to interpret CNV calling from, we have established an approach, using the PennCNV-Affy methodology, to robustly define the reference diploid state and extract not only informative intensity data but also relative genotype content, optimized HMM, and careful review of CNV calls, loci recurrently impacted by CNVs, raw BAF and LRR values, and raw Cartesian coordinates all support that these CNVs are real biological phenomenon. We have experimentally validated a large number of Perlegen-generated CNV calls with the independent method qPCR and demonstrated their inheritance through families as a further validation for the CNV calling [9]. The association of 5q35.1 and underlying CNV calls are robust as evidenced by the nearly 200 SNP probes in agreement of duplication in these five unrelated MDD samples.

We found that the first 4 exons of *SLIT3* (slit homolog 3), the entire *CCDC99* gene and the first 15 exons of *DOCK2* were impacted by the duplication in all cases. Based on functional classifications of these genes, *SLIT3* is the strongest functional candidate due to its role in axon development. *SLIT3* is integral to repulsive axon guidance based on binding to Roundabout receptors. Slits are large, secreted repulsive axon guidance molecules which function as ligands for Roundabout (Robo) receptors. *Slit3* has been characterized as an important player in the genesis of the diaphragm and kidney. Slit3 promotes angiogenesis, a process essential for proper organogenesis during embryonic development [10]. Slits and Robo receptors have been observed to be silenced in various cancer types, implicating its role as a tumor suppressor. *CCDC99* (coiled-coil domain containing 99) was predicted to be a mitotic spindle protein, suggested to be a human homologue of Drosophila Spindly, and proven to localize at the spindle pole by immunofluorescence microscopy [11]. *DOCK2* (dedicator of cytokinesis 2) encodes a protein specific to hematopoietic cells and is indispensable for lymphocyte migration by triggering cytoskeleton reorganization via Rac activation [12]. Thus, the most robustly detected CNV common to the most MDD cases and exclusive when compared to a large control cohort typed on the same platform impacts *SLIT3*, an axon guidance gene.

The controls collected within the MDD study (n = 1,697) were screened based on never scoring high (>0.65) on a general factor score for anxious depression and never reported a history of MDD in any survey, whereas the psoriasis (n = 1,600) and ADHD (n = 1,209) control supplement lacked MDD screening in the controls. Therefore, MDD cases could exist within the controls where they could mask true association signals. Assuming the population frequency of MDD at 16% [13], an estimated 449 subjects within the psoriasis and ADHD projects may have MDD yielding up to 10% of the total control with MDD. These controls were used nonetheless to provide the best matched genotyping platform controls available.

We sought to evaluate the specific phenotypes of the 5 MDD cases with the duplication of the *SLIT3* locus. Although 77 clinical parameters were available for these individuals, no clear bias for specific co-morbidities was evident. The age of onset for depression symptoms was 8, 16, 33, 30, and 30. Two were males and three were females. Interestingly, the total scores for Neuroticism Extraversion Openness (NEO) five-factor inventory short form neuroticism were notably elevated at an average of 47 whereas the MDD population average was 34. Additional characteristics including items from NEO, Inventory of Depression Severity (IDS) depression, and Beck Anxiety Inventory (BAI) anxiety are included in Table S1.

We were unable to acquire the 5 samples to validate the presence of the *SLIT3* locus duplication with an independent technology such as qPCR, FISH, or MLPA. Without this data, the possibility of false positives exists, albeit minimal from our experience. 83 SNPs showed AAB or ABB genotypes which are only possible in duplications throughout 198 SNP probe signals in agreement of the duplication signal with physical size 646 kb. We similarly used conservative inclusion thresholds to mitigate the false positives from this array. Parent and sibling samples were not collected or available to determine if these duplications were inherited but family history of MDD was reported by all 5 individuals.

We conducted a large scale CNV study on 1,693 MDD cases and 4,506 controls typed on the Perlegen 600 K platform. This report represents the first successful CNV analysis using the Perlegen 600 K platform and the first scan for CNVs contributing to MDD susceptibility. CNVs were associated to MDD based on the frequency in cases and exclusivity when compared to controls. The most significant locus included a 646 kb duplication of *SLIT3*, *CCDC99*, and *DOCK2* on 5q35.1 shared by 5 MDD cases. These results offer a highly penetrant variation which underlies MDD involving axon structure and guidance, thereby extending previous observations involving neurotransmitter actions as the underlying cause of MDD.

## Methods

### Ethics Statement

This research was approved by the institutional review board or the Children's Hospital of Philadelphia. Informed consent from the participants was obtained and data were analyzed with anonymous encrypted labels. Written consent was given by the patients for their information to be stored in the hospital database and used for research.

### Case:Control Data

Raw genotyping data from three Genetic Association Information Network (GAIN) [14] projects typed on the Perlegen 600 K (Perlegen Sciences Mountain View, CA, USA) array were accessed through dbGaP. MDD cases and controls who were at low liability for MDD were utilized from the case:control project “Major Depression: Stage 1 Genomewide Association in Population-Based Samples (phs000020.v2.p1)”. Psoriasis Cases and Controls were used to supplement our Perlegen 600 K control cohort for MDD “Collaborative Association Study of Psoriasis (phs000019.v1.p1)”. Lastly, parents from parent-offspring trios were used to further supplement the control from “International Multi-Center ADHD Genetics Project (phs000016.v2.p2)”. Parents from the ADHD study were used to maximize the number of unrelated individuals that could be leveraged for optimal study power.

### Case selection

MDD cases were recruited through mental health care organizations, general practices and in the community setting as previously described [15]. The inclusion criteria for the 1,780 (1,693 of which were used in this study) participants are: 1) a DSM-IV diagnosis of major depressive disorder as confirmed by the CIDI psychiatric interview, 2) an age between 18 through 65 years, 3) sufficient knowledge of the Dutch language, and 4) North-European ancestry. As the samples should be representative of patients seen in different settings, there are few a priori exclusion criteria. Excluded patients are: 1) those with a primary diagnosis of psychosis, bipolar disorder, obsessive compulsive disorder, severe addiction disorder and 2) those with insufficient knowledge of the Dutch language.

### Control selection

Control subjects matched for age and gender were mainly derived from the Netherlands Twin Register, for which data collection in twins, their parents, spouses and siblings occurred in 1991, 1993, 1995, 1997, 2000, 2002/3 and 2004/5. A total of 1860 (1,697 of which were used in this study) controls were selected (only one member from each family) with the following inclusion criteria: 1) age 18 through 65 years, 2) never scoring high (>0.65) on a general factor score for anxious depression (a combined measure of neuroticism, anxiety and depressive symptoms via questionnaires [15]), 3) never reported a history of MDD in any survey, and 4) North-European ancestry. Controls and their parents were born in the Netherlands or northwestern Europe.

Additional control subjects were obtained from two other studies both of which were unrelated to MDD. The first one included a case control study on psoriasis who were genotyped on the Perlegen platform and included as controls (n = 1,600). The psoriasis cases were diagnosed by dermatologists and their matched controls had no history of psoriasis, no family history of psoriasis or other auto-immune disorders. All subjects were 18 years of age or older. The second control cohort included parents from the ADHD parent-offspring trios study who were also genotyped on the Perlegen platform and included as controls (n = 1,209).

Additional controls on the Illumina platform were typed on the We performed high-throughput genome-wide SNP genotyping using the InfiniumII HumanHap550 BeadChip technology (Illumina San Diego CA), at the Center for Applied Genomics at CHOP. Subjects were primarily recruited from the Philadelphia region through the Hospital's Health Care Network, including four primary care clinics and several group practices and outpatient practices that performed well child visits. Eligibility criteria for this study included all of the following: (1) disease-free children and parents of these children in the age range of 0–18 yr of age who had high quality, genome-wide genotyping data from blood samples (defined in Supplemental Methods); (2) self-reported ethnic background; and (3) no serious underlying medical disorder, including but not limited to neurodevelopmental disorders, cancer, chromosomal abnormalities, and known metabolic or genetic disorders.

### PennCNV-Affy Workflow Adapted to Perelgen 600 K Data

The CNV calling on the Perlegen platform used a highly similar algorithm to those used on the Illumina arrays, but the signal pre-processing steps differ. Unlike the Illumina platform, where normalized signal intensities (Log R Ratio and B Allele Frequency) can be exported directly from the BeadStudio software, these signal intensity measures in the Perlegen 600 K platform need to be calculated from the collection of genotyped samples based on raw X and Y values. To perform data normalization and signal extraction from raw final report files generated in genotyping experiments, we first reformatted data from dbGaP into the format produced by Affymetrix Power Tools: birdseed.calls.txt, birdseed.confidences.txt, and quant-norm.pm-only.med-polish.expr.summary.txt. The X and Y values provided in the sample based report files from dbGaP were reduced to a smaller range by taking the logarithm base 10. For each SNP marker, we then relied on the allele-specific signal intensity for the AA, AB and BB genotypes on all genotyped samples to construct three canonical genotype clusters in polar coordinates theta and R, similar to the Illumina clustering generation approach. The “-conf 2” option was included in running generate_affy_geno_cluster.pl since 1 was coded as the best score. Once the canonical genotype clusters were constructed, we then transformed the signal intensity values for each SNP to Log R Ratio (LRR) and B Allele Frequency (BAF) values using normalize_affy_geno_cluster.pl. For more technical details, see http://www.openbioinformatics.org/penncnv/penncnv_tutorial_affy_gw6.html.

To optimize the Hidden Markov Model (HMM), we used the baseline reference file HH550.hmm and ran “-train” in PennCNV^10^ in three successive batches of thirty. The first training used the samples with the lowest standard deviation of LRR while the other two runs, using the file created as a new reference, included more random representative samples. We also created definition files providing inter-SNP distance and population b-allele frequency to further inform CNV calling specifically adapted to the observed Perlegen data. This allowed for CNV calls to be made with good quality metric scores in 6,199 out of 8,231 Perlegen 600 K samples available. Although the global standard deviation of LRR was below 0.2 for the majority (84%) of samples, the intensity data was notably noisier and often showed a subpopulation of SNPs unable to differentiate a deletion signal, perhaps due to PCR saturation during the lab processing. Nevertheless, the deletion and duplication features were still detected with confirmation of homozygote and AAB/ABB genotypes respectively shown for the same SNPs. The SNP level data underlying each CNV call was reviewed to ensure clean signal quality (Figure 2). To ensure that each detected CNV was a true DNA feature and not in any way an artifact of the Perlegen 600 K array used or our bioinformatics manipulations of the data, we report only CNV calls with at least 10 SNPs and 100 kb and reviewed the raw Cartesian plots for underlying SNPs (Figure 3).

### CNV quality control criteria

We calculated Quality Control (QC) measures on our Perlegen 600 K GWAS data based on statistical distributions to exclude poor quality DNA samples and false positive CNVs. The first threshold is the percentage of attempted SNPs which were successfully genotyped. Only samples with call rate >98% were included. The genome wide intensity signal must have as little noise as possible. Only samples with the standard deviation (SD) of normalized intensity (LRR) <0.25 were included. All samples must have Caucasian ethnicity based on Eigenstrat principle components analysis of genotypes and all other samples were excluded. Wave artifacts roughly correlating with GC content resulting from hybridization bias of low full length DNA quantity are known to interfere with accurate inference of copy number variations [16]. Only samples where the GC corrected wave factor of LRR ranged between −0.02<X<0.02 were accepted. If the count of CNV calls made by PennCNV exceeds 100, the DNA quality is usually poor. Thus, only samples with CNV call count <100 were included. Any duplicate samples (such as monozygotic twins) had one sample excluded.

### Statistical analysis of CNVs

CNV frequency between cases and controls was evaluated at each SNP using Fisher's exact test. We only considered loci where cases in the MDD cohort had the same variation and were not observed in any of the control subjects. We report statistical local minimums to narrow the association in reference to a region of nominal significance including SNPs residing within 1 Mb of each other. Resulting nominally significant CNVRs were excluded if they met any of the following criteria: i) residing on telomere or centromere proximal cytobands; ii) arising in a “peninsula” of common CNV arising from variation in boundary truncation of CNV calling; iii) genomic regions with extremes in GC content which produces hybridization bias; or iv) samples contributing to multiple CNVRs.

## Supporting Information

Table S1 Specific phenotypes of the 5 MDD cases with the duplication of the *SLIT3* locus to evaluate co-morbidities
(DOC)

## References

[1] American Psychiatric Association (1994). Diagnostic and Statistical Manual of Mental Disorders..

[2] Glessner JT, Wang K, Cai G, Korvatska O, Kim CE (2009). Autism genome-wide copy number variation reveals ubiquitin and neuronal genes.. Nature 459,.

[3] Wang K, Zhang H, Ma D, Bucan M, Glessner JT (2009). Common genetic variants on 5p14.1 associate with autism spectrum disorders.. Nature.

[4] Sullivan PF, de Geus EJ, Willemsen G, James MR, Smit JH (2008). Genome-wide association for major depressive disorder: a possible role for the presynaptic protein piccolo.. Mol Psychiatry.

[5] Shyn SI, Shi J, Kraft JB, Potash JB, Knowles JA, Weissman MM (2009). Novel loci for major depression identified by genome-wide association study of Sequenced Treatment Alternatives to Relieve Depression and meta-analysis of three studies.. Mol Psychiatry. [Epub ahead of print].

[6] McMahon FJ, Akula N, Schulze TG, Muglia P, Bipolar Disorder Genome Study (BiGS) Consortium (2010). Meta-analysis of genome-wide association data identifies a risk locus for major mood disorders on 3p21.1.. Nat Genet.

[7] Muglia P, Tozzi F, Galwey NW, Francks C, Upmanyu R (2008). Genome-wide association study of recurrent major depressive disorder in two European case-control cohorts.. Mol Psychiatry. [Epub ahead of print].

[8] Wang K, Li M, Hadley D, Liu R, Glessner J (2007). PennCNV: an integrated hidden Markov model designed for high-resolution copy number variation detection in whole-genome SNP genotyping data.. Genome Res.

[9] Hakonarson H, Glessner JT, Wang K, Gai X, Takahashi N (2009). Genome Wide Copy Number Variation Study Associates Metabotropic Glutamate Receptor Genes with Attention Deficit Hyperactivity Disorder. (abstract/program # 2741).. http://www.ashg.org/2009meeting/abstracts/fulltext/.

[10] Zhang B, Dietrich UM, Geng JG, Bicknell R, Esko JD (2009). Repulsive axon guidance molecule Slit3 is a novel angiogenic factor.Blood.. 2009 Nov 5;.

[11] Chan YW, Fava LL, Uldschmid A, Schmitz MH, Gerlich DW (2009). Mitotic control of kinetochore-associated dynein and spindle orientation by human Spindly. J Cell Biol.. 2009 Jun 1;.

[12] Fukui Y, Hashimoto O, Sanui T, Oono T, Koga H (2001). Haematopoietic cell-specific CDM family protein DOCK2 is essential for lymphocyte migration.. Nature.

[13] Kessler RC, Berglund P, Demler O, Jin R, Koretz D, Merikangas KR, Rush AJ, Walters EE, Wang PS National Comorbidity Survey Replication. The epidemiology of major depressive disorder: results from the National Comorbidity Survey Replication (NCS-R). JAMA.. 2003 Jun 18;.

[14] Manolio TA, Rodriguez LL, Brooks L, Abecasis G, Ballinger D (2007). New models of collaboration in genome-wide association studies: the Genetic Association Information Network.. Nat Genet.

[15] Boomsma DI, Willemsen G, Sullivan PF, Heutink P, Meijer P (2008). Genome-wide association of major depression: description of samples for the GAIN Major Depressive Disorder Study: NTR and NESDA biobank projects.. Eur J Hum Genet.

[16] Diskin S, Li M, Hou C, Yang S, Glessner J (2008). Adjustment of genomic waves in signal intensities from whole-genome SNP genotyping platforms.. Nucleic Acids Research.

