# Supplementary material for: Duplication of the SLIT3 Locus on 5q35.1 Predisposes to Major Depressive Disorder
Source: PLoS One. 2010 Dec 1;5(12):e15463. doi: 10.1371/journal.pone.0015463 (PMC2995745; doi:10.1371/journal.pone.0015463)
Supplement: Table S1 — Specific phenotypes of the 5 MDD cases with the duplication of the SLIT3 locus to evaluate co-morbidities (DOC) [file pone.0015463.s001.doc]

Table S1. Specific phenotypes of the 5 MDD cases with the duplication of the *SLIT3* locus to evaluate co-morbidities

| dbGaP Subject-ID | 5533 | 6405 | 6891 | 6903 | 8580 | Description |
| --- | --- | --- | --- | --- | --- | --- |
| ID | 05D01518 | 06D02197 | 06D06042 | 06D06073 | 06D11362 | Case identifier (based on DNA sample code) |
| Source | 0 | 0 | 0 | 0 | 0 | Source of case 0=The Netherlands Study of Depression and Anxiety (NESDA) |
| Case | 1 | 1 | 1 | 1 | 1 | Is this participant a case or a control? 1=case |
| Age | 26 | 40 | 47 | 37 | 48 | Age at most recent assessment |
| Sex | 2 | 1 | 2 | 1 | 2 | Sex of participant 1=Male, 2=Female |
| European | 1 | 1 | 1 | 1 | 1 | North-European ancestry 1=born in Netherlands |
| Education | 2 | 3 | 2 | 1 | 2 | Highest education finished with a diploma 1=low educational level (through lower vocational education), 2=middle educational level (general intermediate through intermediate vocational education), 3=high educational level (higher secondary education or higher) |
| Marital status | 0 | 1 | 1 | 1 | 1 | Marital/partner status 0=no partner and not currently married, 1=currently married or having a partner |
| MDD | 1 | 1 | 1 | 1 | 1 | MDD CIDI-diagnosis - presence 1=yes |
| MDD age | 8 | 16 | 33 | 30 | 30 | MDD CIDI-diagnosis - age of onset |
| Smoker | 0 | 1 | 0 | 0 | 0 | Do you currently smoke cigarettes, pipe of other tobacco? 0=not currently smoking, 1=currently smoking |
| Alcohol | 0 | 1 | 1 | 1 | 1 | In past 12 months, did you have at least 12 alcoholic drinks? 0=no drinker, 1=drinker |
| #MDD episodes | 1 | 1 | 2 | 1 | 3 | Number of mdd episodes lifetime |
| NEOneu1 | 2 | 1 | 1 | 5 | 2 | NEO-neuroticism item 1 |
| NEOneu2 | 4 | 3 | 5 | 4 | 2 | NEO-neuroticism item 2 |
| NEOneu3 | 4 | 4 | 4 | 3 | 4 | NEO-neuroticism item 3 |
| NEOneu4 | 5 | 1 | 1 | 3 | 3 | NEO-neuroticism item 4 |
| NEOneu5 | 4 | 4 | 3 | 5 | 4 | NEO neuroticism item 5 |
| NEOneu6 | 4 | 4 | 5 | 4 | 4 | NEO neuroticism item 6 |
| NEOneu7 | 2 | 1 | 1 | 1 | 2 | NEO neuroticism item 7 |
| NEOneu8 | 3 | 3 | 4 | 5 | 3 | NEO neuroticism item 8 |
| NEOneu9 | 4 | 4 | 5 | 3 | 2 | NEO neuroticism item 9 |
| NEOneu10 | 2 | 1 | 1 | 2 | 2 | NEO neuroticism item 10 |
| NEOneu11 | 4 | 4 | 5 | 4 | 3 | NEO neuroticism item 11 |
| NEOneu12 | 4 | 3 | 5 | 4 | 4 | NEO neuroticism item 12 |
| neoneu | 44 | 49 | 56 | 45 | 41 | NEO-FFI short form Neuroticism: Total scale score |
| ids01 | 2 | 0 | 0 | 2 | 1 | Inventory of Depression Severity (IDS) depression item 1: Falling asleep |
| ids02 | 1 | 1 | 3 | 3 | 0 | Inventory of Depression Severity (IDS) depression item 2: Sleep during the night |
| ids03 | 3 | 0 | 0 | 0 | 0 | Inventory of Depression Severity (IDS) depression item 3: Waking up too early |
| ids04 | 1 | 0 | 0 | 1 | 0 | Inventory of Depression Severity (IDS) depression item 4: Sleeping too much |
| ids05 | 2 | 3 | 1 | 3 | 1 | Inventory of Depression Severity (IDS) depression item 5: Feeling sad |
| ids06 | 0 | 2 | 2 | 3 | 2 | Inventory of Depression Severity (IDS) depression item 6: Feeling irritable |
| ids07 | 2 | 2 | 0 | 2 | 1 | Inventory of Depression Severity (IDS) depression item 7: Feeling anxious or tense |
| ids08 | 1 | 0 | 0 | 2 | 0 | Inventory of Depression Severity (IDS) depression item 8: Response of your mood to good or desired events |
| ids09a | 0 | 0 | 3 | 0 | 3 | Inventory of Depression Severity (IDS) depression item 9a: Mood in relation to the time of the day |
| ids09b | -1 | -1 | 2 | -1 | 1 | Inventory of Depression Severity (IDS) depression item 9b: Is your mood typically worse in the: |
| ids09c | -1 | -1 | 1 | -1 | 2 | Inventory of Depression Severity (IDS) depression item 9c: Is your mood variation attributed to the environment? |
| ids10 | 2 | 3 | 1 | 0 | 0 | Inventory of Depression Severity (IDS) depression item 10: The quality of your mood |
| ids11 | 5 | 1 | 7 | 2 | 1 | Inventory of Depression Severity (IDS) depression item 11: Changed Appetite |
| ids12 | 5 | 1 | 6 | 2 | 1 | Inventory of Depression Severity (IDS) depression item 12: Changes in weight (within the last two weeks) |
| ids13 | 2 | 2 | 2 | 3 | 1 | Inventory of Depression Severity (IDS) depression item 13: Concentration/Decision Making |
| ids14 | 1 | 3 | 2 | -1 | 0 | Inventory of Depression Severity (IDS) depression item 14: View of Myself |
| ids15 | 1 | 2 | 1 | 1 | 0 | Inventory of Depression Severity (IDS) depression item 15: View of my future |
| ids16 | 2 | 2 | 2 | 0 | 2 | Inventory of Depression Severity (IDS) depression item 16: Thoughts of Death or Suicide |
| ids17 | 2 | 0 | 1 | 3 | 0 | Inventory of Depression Severity (IDS) depression item 17: General Interest |
| ids18 | 2 | 2 | 1 | 3 | 2 | Inventory of Depression Severity (IDS) depression item 18: Energy Level |
| ids19 | 1 | 2 | 1 | 3 | 0 | Inventory of Depression Severity (IDS) depression item 19: Capacity for Pleasure or Enjoyment (excluding sex) |
| ids20 | 2 | 0 | 3 | 1 | 1 | Inventory of Depression Severity (IDS) depression item 20: Interest in Sex (Please Rate Interest, not Activity) |
| ids21 | 2 | 0 | 2 | 2 | 0 | Inventory of Depression Severity (IDS) depression item 21: Feeling slowed down |
| ids22 | 2 | 0 | 0 | 2 | 0 | Inventory of Depression Severity (IDS) depression item 22: Feeling restless |
| ids23 | 2 | 2 | 1 | 2 | 2 | Inventory of Depression Severity (IDS) depression item 23: Aches and pains |
| ids24 | 1 | 0 | 0 | 3 | 1 | Inventory of Depression Severity (IDS) depression item 24: Other bodily symptoms |
| ids25 | 2 | 0 | 2 | 1 | 1 | Inventory of Depression Severity (IDS) depression item 25: Panic/Phobic symptoms |
| ids26 | 1 | 0 | 0 | 1 | 1 | Inventory of Depression Severity (IDS) depression item 26: Constipation/diarrhea |
| ids27 | 3 | 1 | 2 | 3 | 3 | Inventory of Depression Severity (IDS) depression item 27: Interpersonal sensitivity |
| ids28 | 2 | 2 | 1 | 3 | 1 | Inventory of Depression Severity (IDS) depression item 28: Leaden paralysis/physical energy |
| ids_tot | 44 | 29 | 36 | 49 | 23 | Inventory of Depression Severity (IDS) Score - Degree of severity of current depressive symptoms |
| bai01 | 0 | 0 | 0 | 3 | 0 | Beck Anxiety Inventory (BAI) anxiety item 1: Numbness or tingling |
| bai02 | 0 | 0 | 1 | 2 | 2 | Beck Anxiety Inventory (BAI) anxiety item 2: Feeling hot |
| bai03 | 2 | 0 | 0 | 1 | 0 | Beck Anxiety Inventory (BAI) anxiety item 3: Wobbliness in legs |
| bai04 | 1 | 1 | 2 | 3 | 1 | Beck Anxiety Inventory (BAI) anxiety item 4: Unable to relax |
| bai05 | 2 | 0 | 3 | 2 | 1 | Beck Anxiety Inventory (BAI) anxiety item 5: Fear of worst happening |
| bai06 | 1 | 0 | 1 | 3 | 0 | Beck Anxiety Inventory (BAI) anxiety item 6: Dizzy or light-headed |
| bai07 | 1 | 0 | 0 | 2 | 1 | Beck Anxiety Inventory (BAI) anxiety item 7: Heart pounding/racing |
| bai08 | 1 | 0 | 0 | 3 | 0 | Beck Anxiety Inventory (BAI) anxiety item 8: Unsteady |
| bai09 | 0 | 0 | 0 | 1 | 0 | Beck Anxiety Inventory (BAI) anxiety item 9: Terrified or afraid |
| bai10 | 2 | 1 | 1 | 3 | 2 | Beck Anxiety Inventory (BAI) anxiety item 10: Nervous |
| bai11 | 1 | 0 | 0 | 2 | 0 | Beck Anxiety Inventory (BAI) anxiety item 11: Feeling of choking |
| bai12 | 2 | 0 | 0 | 2 | 0 | Beck Anxiety Inventory (BAI) anxiety item 12: Hands trembling |
| bai13 | 2 | 0 | 1 | 2 | 0 | Beck Anxiety Inventory (BAI) anxiety item 13: Shaky/unsteady |
| bai14 | 2 | 1 | 2 | 3 | 1 | Beck Anxiety Inventory (BAI) anxiety item 14: Fear of losing control |
| bai15 | 0 | 0 | 0 | 2 | 0 | Beck Anxiety Inventory (BAI) anxiety item 15: Difficulty in breathing |
| bai16 | 1 | 0 | 1 | 0 | 0 | Beck Anxiety Inventory (BAI) anxiety item 16: Fear of dying |
| bai17 | 2 | 1 | 3 | 2 | 1 | Beck Anxiety Inventory (BAI) anxiety item 17: Scared |
| bai18 | 1 | 0 | 0 | 2 | 1 | Beck Anxiety Inventory (BAI) anxiety item 18: Indigestion |
| bai19 | 2 | 2 | 1 | 3 | 0 | Beck Anxiety Inventory (BAI) anxiety item 19: Faint/light-headed |
| bai20 | 0 | 0 | 1 | 3 | 2 | Beck Anxiety Inventory (BAI) anxiety item 20: Face flushed |
| bai21 | 0 | 0 | 1 | 2 | 2 | Beck Anxiety Inventory (BAI) anxiety item 21: Hot/cold sweats |
| baiscal | 23 | 6 | 18 | 46 | 14 | Beck Anxiety Inventory (BAI) Total scale score - Degree of current anxiety |
| famrisc | 1 | 1 | 1 | 1 | 1 | Family history of major depression: First degree family member with depression |

Data retrieved from dbGaP datasets pht000070.v1.p1.c1 and pht000667.v1.p1.c1.
